# Une intoxication peut en cacher une autre plus grave. Exemple d'une intoxication fatale à l'éthylène glycol masquée par une intoxication à un insecticide pyréthrinoïde

**DOI:** 10.11604/pamj.2013.14.128.2205

**Published:** 2013-04-02

**Authors:** Younès Aissaoui, Hicham Kichna, Mohammed Boughalem, Noureddine Drissi Kamili

**Affiliations:** 1 Pôle Anesthésie Réanimation, Hôpital Militaire Avicenne, Université Cadi Ayyad, Marrakech; 2 Pôle Anesthésie Réanimation, Hôpital Militaire Mohammed V, Université Mohammed V Souissi, Rabat, Maroc

**Keywords:** Intoxication, insecticide, pyréthrinoïde, cyperméthrine, éthylène glycol, solvant, Intoxication, insecticide, pyrethroid, cypermethrin, ethylene glycol, solvent

## Abstract

Les pyréthrinoïdes sont des insecticides largement utilisés du fait de leur efficacité et de leur relative sécurité chez l'homme. Les intoxications mortelles liées à ces agents restent exceptionnelles. Leur métabolisme hépatique rapide limite considérablement leur toxicité chez l'homme. Cette observation relate une intoxication grave à un pyréthrinoïde (la cyperméthrine) dont le solvant était l'éthylène glycol. Ce dernier est un toxique nettement plus dangereux pour l'homme. Le tableau clinique consistait en une atteinte multiviscérale avec prédominance de la défaillance cardiovasculaire et neurologique. Le faible potentiel toxique des pyréthrinoïdes suggère l'implication évidente de l'éthylène glycol dans la gravité de cette intoxication. La prise en charge thérapeutique, essentiellement symptomatique, n'a pas pris en compte la présence d'éthylène glycol dans la formulation de l'insecticide. L'évolution clinique était défavorable. Devant toute intoxication grave à un insecticide pyréthrinoïde une intoxication associée au solvant tel que l'éthylène glycol doit être recherchée et traitée.

## Introduction

Les pyréthrinoïdes constituent les insecticides les plus souvent employés en usage agricole, vétérinaire et domestique. En effet, ils représentent plus de la moitié du marché mondial des insecticides et sont largement utilisés en Afrique [1]. Cette large utilisation s'explique par leur grande efficacité sur les insectes et leur relative sécurité chez l'homme [2]. Les intoxications fatales aux pyréthrinoïdes sont exceptionnelles dans la littérature médicale [3, 4]. Dans certaines de ces intoxications, l'exposition aux pyréthrinoïdes s'accompagne d'une exposition concomitante à un solvant organique, qui fait partie de la formulation chimique de l'insecticide. Le potentiel toxique du solvant peut être plus grave que l'insecticide lui-même. Le cas clinique que nous rapportons illustre bien ce fait. Il s'agissait d'une intoxication mortelle à l'éthylène glycol due à l'ingestion d'un insecticide à base de cyperméthrine [5].

## Patient et observation

Un patient de 27 ans, sans antécédents pathologiques, a été admis dans notre formation environ dix heures après ingestion orale d'un litre d'un insecticide, dans un but d'autolyse. Une lettre expliquant qu'un conflit familial l'aurait poussé à se suicider a été retrouvée dans son domicile. Le flacon contenant l'insecticide n'a pas été acheminé avec le patient. Il nous a été communiqué par téléphone qu'il s'agissait d'un pyréthrinoïde: la cyperméthrine.

Son examen clinique trouvait une pression artérielle à 70/40 mmHg, une bradycardie à 40/min, des marbrures généralisées, une turgescence des veines jugulaires et une hypothermie à 35.5°c. Sur le plan ventilatoire, le patient était bradypneique avec une fréquence respiratoire à 10/min, cyanosé et à l'auscultation des champs pulmonaires libres. Le signal de plethysmographie n'était pas perceptible. L'examen neurologique trouvait un coma calme, profond, (score de Glascow = 3) avec des pupilles en mydriase légèrement réactives.

Le patient a été immédiatement admis en réanimation. Un remplissage vasculaire a été entrepris (1000 ml de sérum physiologique et 1000 ml de plasmion^®^) avec administration concomitante d'atropine et d'éphédrine. Devant l'inefficacité de plusieurs boli d'atropine (3 x 1mg) et d'éphédrine (5 x 6mg), une voie veineuse centrale (sous clavière) a rapidement été mise en place avec perfusion continue d'adrénaline. La pression veineuse centrale mesurée était de 20 mmHg. Par ailleurs, le patient a été intubé et mis sous ventilation artificielle. Une sonde nasogastrique à double courant a été mise en place et n'a rien ramené à l'aspiration. Il n'a pas été effectué de lavage gastrique. Le cathétérisme

**Figure 1:**
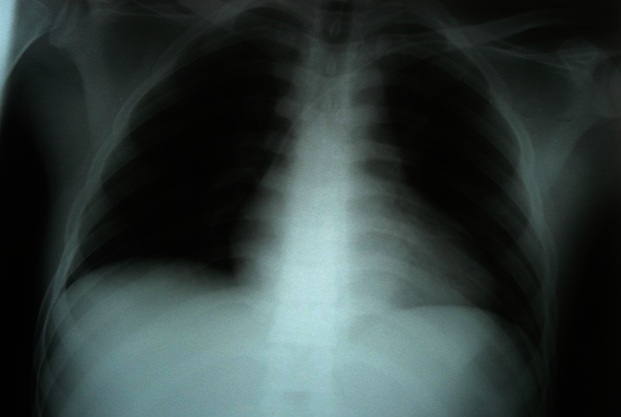
Radiographie du thorax chez un patient ayant présenté une intoxication massive à la cyperméthrine et à l'éthylène glycol. Elle montre la présence d'une silhouette cardiaque augmentée de taille témoignant de l'atteinte cardiaque toxique.

**Figure 2:**
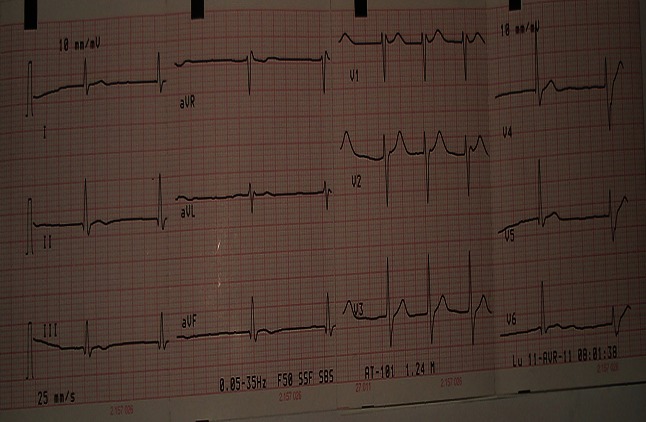
électrocardiogramme montrant la paralysie sinusale (notez l'absence des ondes p) avec un rythme d'échappement jonctionnel.

La radiographie du thorax a montré une silhouette cardiaque augmentée de taille avec une transparence pleuro-pulmonaire normale (Figure 1). L'électrocardiogramme montrait une paralysie sinusale avec un échappement jonctionnel à une fréquence de 40/min, un bloc de branche droit incomplet et des extrasystoles ventriculaires (Figure 2). L'échocardiographie transthoracique a montré une dilatation des cavités cardiaques avec un effondrement de la contractilité myocardique (hypokinésie globale avec fraction d'éjection à 20%). La tomodensitométrie cérébrale sans injection de contraste et réalisé après stabilisation hémodynamique était normale. Le bilan biologique réalisé comprenait: hémogramme, hémostase, ionogramme sanguin, urée et créatinine, glycémie, bilan hépatique (transaminases, bilirubine, phosphatases alcalines), enzymes musculaires (CPK et CPK_MB_), Troponine, gaz du sang et prélèvement toxicologiques. Les anomalies observées étaient une hyperleucocytose à 13000/mm^3^, une élévation de la créatinine sanguine à 24 mg/l et une acidose métabolique à trou anionique élevée (pH = 7.15 et HCO_3_ = 12 meq/l et trou anionique = 32 meq/l). De ce fait, le patient a fait l'objet d'une alcalinisation par bicarbonate 4.2% 500 ml en perfusion dans l'objectif d'améliorer l'efficacité de l'adrénaline administrée. Le patient est décédé après 12 heures d'admission en réanimation par état de choc réfractaire.

Les prélèvements toxicologiques réalisés (sang, urines et liquides gastriques) étaient négatifs. Ce screening toxicologique comprenait la recherche de médicaments (benzodiazépines, barbituriques, imipramines, chloral) et de pesticides (carbamates, chloralose, organophosphorés, organochlorés, coumariniques) Après décès du patient, nous avons reçu le flacon de l'insecticide incriminé. Il s'agissait de cyperméthrine dosée à 10% (Alphamost 10 SC^®^). La dose supposé ingérée était de 100 grammes. En consultant, la composition du produit sur internet, nous avons trouvé que le solvant utilisé pour ce pyréthrinoïde était de l'éthylène glycol à 5%. La dose supposée ingérée d'éthylène glycol était donc de 50 ml. Nous avons également adressé un échantillon d'insecticide au centre antipoison afin de rechercher la présence d'organophosphorés. La recherche était négative.

## Discussion

Cette observation relate une intoxication mortelle à la cyperméthrine, après ingestion orale d'une dose importante de ce pyréthrinoïde (100 g). La réputation d'innocuité dont bénéficie cette famille d'insecticides- classés comme modérément dangereux par l'Organisation Mondiale de la Santé [2] - nous a poussés à rechercher une explication à la gravité du tableau clinique observé. Nous avons trouvé que le solvant utilisé pour cet insecticide était l'éthylène glycol. Ceci qui suggère très fortement que la symptomatologie observée et la mortalité de cette intoxication est imputable à l'éthylène glycol plutôt qu'à la cyperméthrine. La cyperméthrine fait partie des pyréthrinoïdes de synthèse qui sont dérivé des pyréthrines naturelles. Ces dernières sont extraites de la fleur jaune de *Chrysanthemum Cinerariaefolium* [1]. Le système nerveux est la principale cible des pyréthrinoïdes qui agissent en prolongeant l'ouverture des canaux sodiques membranaires. Le système nerveux des insectes est des milliers de fois plus sensible aux pyréthrinoïdes que celui des mammifères, ce qui explique leur innocuité chez ces derniers et leur excellente efficacité insecticide [2, 4]. Les pyréthrinoïdes rapidement métabolisés par le foie, ce qui limite leur toxicité systémique. Après ingestion orale, la symptomatologie observée se limite à des douleurs abdominales et à des nausées-vomissements [6]. Il existe deux classes de pyréthrinoïdes. Les composés du groupe 1 (sans groupe cyano) et les composés du groupe 2 (avec groupe cyano) [2, 4]. Les composés du groupe 2 - dont fait partie la cyperméthrine ‘ sont plus toxiques que ceux du groupe 1. Classiquement, l'intoxication par les dérivés du groupe 1 est responsable d'ataxie, d'augmentation de la sensibilité aux stimuli sensoriels, de tremblements généralisés voire de convulsions (syndrome T). Les dérivés du groupe 2 entrainent une choréo-athétose, une hypersalivation, convulsions et décès par paralysies [1, 2, 4].

L'éthylène glycol est un glycol utilisé comme antigel (liquide de refroidissement pour automobile, circuit de chauffage) et comme solvant industriel [5]. L'intoxication à cet agent et rare mais est très grave. Elle entraine par le biais de la formation de l'acide glycolique, une insuffisance rénale, troubles de conscience, incompétence myocardique et défaillance multivsicérale [5, 7, 8]. Par conséquent, les symptômes observés sont plutôt attribuable à l'intoxication à l'éthylène.

En effet, le patient avait un coma profond expliqué par l'intoxication l'éthylène glycol [5, 7, 8]. La cyperméthrine, à l'inverse engendre un coma agité avec convulsions et chorée-athétose [9]

L'état de choc observé était d'origine cardiogènique comme en témoigne les troubles de conduction cardiaque observés et la baisse globale de la contractilité myocardique retrouvée à l'échocardiographie. Néanmoins, une composante vasculaire (vasoplégie) reste probable comme en témoigne la baisse de la pression artérielle diastolique. Ce qui explique le choix de l'adrénaline comme agent cardio-vasoactif. L'éthylène glycol entraine une atteinte cardiaque (myosite) par précipitation myocardique de cristaux d'oxalate de calcium [5]. Ces derniers étant formés par la précipitation de l'acide glycolique, principal métabolite de l'éthylène glycol dans l'organisme. Par ailleurs, les pyréthrinoïdes ayant un effet sur les canaux sodiques voltages dépendants pourraient être partiellement responsable du trouble de conduction observée (pause sinusale) [4]. La seule observation clinique ayant rapporté des troubles similaires était due à l'ingestion orale de pralléthrine [10]. Cependant, l'évolution était favorable. Seuls certains pyréthrinoïdes- dont la cyperméthrine - auraient un potentiel arythmogène [11].

L'atteinte rénale ainsi que l'acidose métabolique observée (à trou anionique élevé) sont classiques dans l'intoxication à l'éthylène glycol par le biais de la production de l'acide formique. [5, 7, 15]. Un argument simple de confirmation de l'intoxication à l'éthylène glycol, est le calcul du trou osmotique (osmolarité mesurée - osmolarité calculée) [5]. Un trou osmotique élevé est très en faveur d'une intoxication à l'éthylène glycol. Cependant, le laboratoire de biologie de notre hôpital ne dispose pas de la mesure de l'osmolarité par delta cryoscopique. Par contre, le dosage de l'éthylène glycol par chromatographie en phase gazeuse est spécifique, mais de cout élevé et non disponible en routine [15]. De même, le dosage des pyréthrinoïdes par chromatographie en phase gazeuse couplée à la spectrométrie de masse est rarement disponible en pratique et n'as pas d'incidence sur la prise en charge qui est essentiellement symptomatique. Par contre, le screening toxicologique que nous avons effectué avait pour objectif d'éliminer une autre intoxication associée.

D'autres solvants dangereux ont été incriminés dans les intoxications aux pyréthrinoïdes. Dans un cas clinique d'intoxication à la deltaméthrine, la cardiotoxicité du solvant Naphta, qui fait partie de la famille des hydrocarbures, était responsable d'une asystolie réfractaire à la réanimation [13]. D'autres insecticides ont été mélangés de façon frauduleuse avec des pyréthrinoïdes, tels que les organophosphorés et ont été responsables d'intoxications particulièrement graves [14]. Dans cette éventualité, nous avons demandé une recherche d'organophosphorés dans l'insecticide incriminé. Cette dernière, réalisée par le centre antipoison était négative.

Dans notre observation, que la dose supposée ingérée d'éthylène glycol était de 50 ml. Rapportée au poids du patient (60 Kg), elle est l'ordre de 0.83 ml/Kg. Cette dose est une dose mortelle puisque la dose létale chez l'homme est de 1 ml/Kg [2].

La difficulté majeure posée par cette intoxication est l'absence d'indication sur le flacon de l'insecticide de sa composition complète. En effet, ce n'est qu'à postériori en consultant sa composition sur internet, que nous avons pu savoir qu'il contenait de l'éthylène glycol à des doses importantes. Cette indication aurait pu avoir des implications thérapeutiques importantes, à savoir l'instauration d'une épuration extra-rénale précoce voire l'administration d'un antidote: fomépizole de préférence à l'éthanol [7].

## Conclusion

Devant toute intoxication à un insecticide pyréthrinoïde, il faut vérifier sa composition exacte à la recherche d'un solvant tel que l'éthylène glycol. En effet, le solvant peut avoir un potentiel toxique plus grave que l'insecticide lui-même et sa présence pourrait avoir d'importantes implications diagnostiques et thérapeutiques. Par ailleurs, la législation doit imposer aux industriels d'indiquer sur l'étiquetage des insecticides tous les composants potentiellement dangereux pour l'homme. Enfin, les solvants dangereux ne devraient pas être utilisés dans la fabrication de ces insecticides.
